# Comparative study of clinical effects between oblique lumbar interbody fusion (OLIF) and lateral lumbar interbody fusion (LLIF) in the treatment of degenerative disc disease of the lumbar spine

**DOI:** 10.12669/pjms.40.10.9344

**Published:** 2024-11

**Authors:** YiXiao Wang, Yang Song, Yue Ma, Xinyu Sun, Hua Wang

**Affiliations:** 1 YiXiao Wang, Department of Orthopaedics, Affiliated Hospital of Beihua University, Jilin 132011, Jilin, China; 2 Yang Song, Department of Orthopaedics, Affiliated Hospital of Beihua University, Jilin 132011, Jilin, China; 3 Yue Ma, Department of Orthopaedics, Affiliated Hospital of Beihua University, Jilin 132011, Jilin, China; 4 Xinyu Sun Department of Orthopaedics, The Fourth Medical Centre of Chinese PLA, General Hospital, BeiJing 100853, China; 5 Hua Wang, Department of Orthopaedics, Affiliated Hospital of Beihua University, Jilin 132011, Jilin, China

**Keywords:** Degenerative disc disease, Lumbar spine, OLIF, LLIF, Lumbar fusion, Clinical effects

## Abstract

**Objective:**

To compare the clinical effects of oblique lumbar interbody fusion (OLIF) and lateral lumbar interbody fusion (LLIF) in the treatment of degenerative disc disease of the lumbar spine.

**Method:**

This was a retrospective study. The clinical data of 80 patients with lumbar disc degenerative disease who underwent surgery in Affiliated Hospital of Beihua University from May 2018 to May 2023 were selected. The patients were divided into LLIF group and OLIF group according to surgical methods. Compare the 36-Item Short-Form Health Survey(SF-36), Visual Analog Scale(VAS) scores, Oswesterly Disability Index(ODI), Japanese Orthopedic Association(JOA) scores, Cobb angle, and intervertebral height changes between two groups of patients pre- and posttreatment, and evaluate the differences in clinical efficacy and surgical complications.

**Result:**

Postsurgery, the SF-36 score, VAS score, ODI index, and JOA score of two groups were significantly better than presurgery(p<0.05); After three months of treatment, the improvement in OLIF group was better than LLIF group(p<0.05), while there was no statistically significant difference between the two groups at six months postsurgery(p>0.05). Six months postsurgery, the intervertebral space height and Cobb angle of the two groups were significantly improved compared to presurgery (p<0.05), but there was no significant difference between the groups(p>0.05). There was no difference in clinical efficacy and incidence between the two groups(p>0.05).

**Conclusion:**

LLIF and OLIF may be both safe and effective minimally invasive surgical methods for the treatment of degenerative disc disease of the lumbar spine.

## INTRODUCTION

Degenerative disc disease (DDD) is a common degenerative disorder in orthopedic clinical practice. There are various treatment modalities available, particularly regarding the timing and indications for spinal fusion surgery, which remain controversial to date.^1^-^3^ Lumbar interbody fusion (LIF) is an effective and classic surgical approach for treating lumbar DDD. LIF can achieve fusion between vertebral bodies with a low incidence of complications. After surgery, patients experience less pain, faster recovery, and shorter hospital stays.^4^-^7^ There are various surgical approaches for LIF, with transforaminal lumbar interbody fusion (TLIF) and posterior lumbar interbody fusion (PLIF) being the most commonly used in clinical practice. However, these approaches carry a higher risk of nerve injury and can disrupt the paraspinal muscles, leading to severe epidural adhesions that affect surgical outcomes.^8^,^9^

In recent years, oblique lumbar interbody fusion (OLIF) and lateral lumbar interbody fusion (LLIF) have been widely used in clinical practice. However, there are few comparative studies on the clinical outcomes and complications between OLIF and LLIF. Therefore, this study aimed to compare the clinical and radiographic outcomes of these two surgical techniques in the treatment of lumbar DDD, evaluate their specific indications and potential technical risks, and accumulate experience for the clinical application and promotion of these techniques.

## METHODS

A retrospective analysis was conducted on clinical data of patients who underwent surgical treatment for Degenerative disc disease (DDD) at Affiliated Hospital of Beihua University from May 2018 to May 2023, and the 80 patients were divided into LLIF group (n= 40) and OLIF group (n= 40) According to the different surgical methods.

### Ethical Approval:

The study was approved by the Institutional Ethics Committee of Affiliated Hospital of Beihua University (No.:2023-25; date: April 18, 2023), and written informed consent was obtained from all participants.

### Inclusion criteria:

- Patients with discogenic low back pain who did not respond to conservative treatment.
- Age between 18 and 80 years old.
- Patients with Pfirmann score of ≥ 3 for intervertebral disc degeneration;
- Patients with chronic axial pain in the lumbar region aggravated by prolonged standing and/or activity.
- Patients who underwent single-level or two-level surgery.

### Exclusion criteria:

- Patients who underwent surgery involving more than two levels.
- Patients with spinal deformity accompanied by overall sagittal plane imbalance.
- Patients with nerve root pain caused by lumbar disc protrusion or spinal canal stenosis.
- Patients with concomitant fractures.
- Patients with coronary heart disease, diabetes and other basic diseases.

Titanium or PEEK cages were used for fusion, and autogenous iliac bone graft was used to fill the cages. Spinal stability was achieved by placing percutaneous pedicle screws. All patients were treated with cage by strictly trained personnel, and the researchers were proficient in both fixation methods. After the operation, drugs should be administered for anti-infection and pain relief.

Imaging examinations included preoperative and postoperative lumbar spine anteroposterior and lateral X-rays in flexion and extension positions. CT and MRI scans were also performed to assess the condition of the lumbar spine, spinal stability, and local major blood vessels. Both groups were followed-up for six months. All operations were performed by the same group of doctors, which has been described in the text.

### Outcome Measures:

The Medical Outcome Study 36-item Short-Form Health Survey (SF-36)^10^ was used to evaluate the patients’ quality of life before surgery, as well as at three months and six months postoperatively. The visual analogue scale (VAS), Oswestry Disability Index (ODI), and Japanese Orthopaedic Association score (JOA) were used to assess the functional outcomes of the lumbar spine in both groups of patients. The degree of improvement in lumbar spine imaging was evaluated by measuring the Cobb angle and intervertebral height of the fused segment using X-rays taken before surgery and at six months postoperatively.

Intervertebral height was defined as the length of the line connecting the midpoint of the upper endplate of the superior vertebra and the midpoint of the lower endplate of the inferior vertebra. The clinical efficacy at six months after treatment was evaluated according to the MacNab criteria for lumbar spine function: Excellent: pain-free, able to perform normal activities; Good: symptoms relieved, occasional pain, limited activity; Fair: some improvement in function, significant pain, unable to perform normal activities; Poor: no change or worsening of symptoms. The excellent and good rates were calculated as the percentage of patients achieving excellent and good outcomes. The intraoperative and postoperative complications were also compared between the two groups.

### Statistical analysis:

Statistical analysis was performed using SPSS 21.0 software. The sample size is estimated by 95% confidence interval, quantitative data were expressed as mean ± standard deviation (*χ* ®±*S*), and t-tests were used for between-group comparisons, two independent sample t test was used. Qualitative data were expressed as frequencies and percentages (%), and Chi-square tests were used for between-group comparisons. P<0.05 was considered statistically significant.

## RESULT

In the LLIF group, there were 23 male patients and 17 female patients, with an age range of 23 to 72 years old and a mean age of 47.00 ± 11.46 years old. A total of 54 segments were treated, with 4 segments at L1/2, 8 segments at L2/3, 14 segments at L3/4, 16 segments at L4/5, and 12 segments at L5/S1. In the OLIF group, there were 29 male patients and 11 female patients, with an age range of 25 to 71 years old and a mean age of 47.48 ± 11.93 years old. A total of 60 segments were treated, with 5 segments at L1/2, 7 segments at L2/3, 15 segments at L3/4, 18 segments at L4/5, and 15 segments at L5/S1. There were no significant differences in general information between the two groups (p>0.05), indicating comparability, Table-I.

**Table-I:** General information of patients in the two groups.

	LLIF Group	OLIF Group	t/χ2	P
Age (years)	47.00±11.46	47.48±11.93	0.182	0.856
Gender (Male/Female)	23/17	29/11	1.978	0.160
Duration of Symptoms (years)	14.13±1.49	13.73±1.81	1.079	0.284
Weight (Ksg)	82.76±3.50	83.04±3.27	0.367	0.715
Treated Segments			0.348	0.986
L1/2	4	5		
L2/3	8	7		
L3/4	14	15		
L4/5	16	18		
L5/S1	12	15		

There was no statistically significant difference in preoperative SF-36 scores between the two groups (p>0.05). Postoperatively, both groups showed significant improvement in SF-36 scores compared to preoperative scores (p<0.05). At three months postoperatively, the OLIF group showed a significantly greater improvement in SF-36 scores compared to the LLIF group (p<0.05), while at the final follow-up, there was no statistically significant difference in SF-36 scores between the two groups (p>0.05), Table-II.

**Table-II:** Comparison of SF-36 Scores before and after Surgery in the Two Groups (*χ* ®±*S*).

Group	Preoperative	3 Months Postoperative	6 Months Postoperative
LLIF Group	51.88±0.76	60.28±1.11	80.00±1.48
OLIF Group	52.03±0.53	66.30±2.31	80.23±1.35
*t*	1.026	14.862	0.709
*P*	0.308	0.000	0.480

There was no statistically significant difference in preoperative VAS scores, ODI index, and SF-36 scores between the two groups(p>0.05). Postoperatively, both groups showed significant reductions in VAS scores and ODI index, and significant improvements in JOA scores compared to preoperative values(p<0.05). At three months postoperatively, the OLIF group demonstrated a significantly greater improvement in VAS scores, ODI index, and SF-36 scores compared to the LLIF group(p<0.05), while at six months postoperatively, there were no statistically significant differences in VAS scores, ODI index, and SF-36 scores between the two groups (p>0.05), Table-III.

**Table-III:** Comparison of VAS Scores, ODI Index, and JOA Scores before and after Surgery in the Two Groups (*χ* ±*S*).

VAS Scores				ODI Index			JOA Scores		

Group	Preoperative	3 Months Postoperative	6 Months Postoperative	Preoperative	3 Months Postoperative	6 Months Postoperative	Preoperative	3 Months Postoperative	6 Months Postoperative
LLIF Group	6.18±0.81	3.45±0.50	0.60±0.50	42.73±1.20	22.08±1.37	2.23±0.58	7.98±0.80	20.03±1.33	24.90±1.37
OLIF Group	6.20±0.56	2.23±0.42	0.50±0.51	42.68±1.29	21.50±1.18	2.13±0.33	7.70±0.88	20.98±1.23	25.03±1.12
*t*	0.160	11.778	0.892	0.180	2.017	0.948	1.460	3.317	0.446
*P*	0.873	0.000	0.375	0.858	0.047	0.346	0.148	0.001	0.657

There was no statistically significant difference in preoperative intervertebral height and Cobb angle between the two groups (p>0.05). At six months postoperatively, both groups showed significant improvements in intervertebral height and Cobb angle compared to preoperative values (p<0.05), but there was no statistically significant difference between the groups (p>0.05), Table-IV.

**Table-IV:** Comparison of Lumbar Radiographic Parameters between the Two Groups (*χ* ®±*S*).

Group	Intervertebral Height (mm)		Cobb angle (º)	

	Preoperative	6 Months Postoperative	Preoperative	6 Months Postoperative
LLIF Group	7.28±0.84	10.55±0.55	8.27±0.32	12.42±0.51
OLIF Group	7.13±0.72	10.84±0.84	8.36±0.32	12.52±0.29
*t*	0.848	1.846	1.196	1.020
*P*	0.399	0.069	0.235	0.311

At six months postoperatively, the excellent and good rates of surgical outcomes in the OLIF group was 92.50%, which was significantly higher than the 77.50% in the LLIF group, but the difference was not statistically significant (p>0.05), Table-V.

**Table-V:** Comparison of Clinical Outcomes between the Two Groups [*n*, (%)].

Group	Excellent	Good	Fair	Poor	The excellent and good rate
LLIF Group	22 (55.00)	9 (22.50)	7 (17.50)	2 (5.00)	31 (77.50)
OLIF Group	25 (62.50)	12 (30.00)	2 (5.00)	1 (2.50)	37 (92.50)
*χ*²					3.529
*P*					0.060

The OLIF group had seven cases of complications, including four cases of transient nerve injury, two cases of dural laceration during surgery, and one case of left iliac vein rupture during surgery. The LLIF group had 11 cases of complications, including two cases of intervertebral space infection, four cases of dural laceration during surgery, three cases of nerve root injury, and one case each of peritoneal laceration and lower limb venous thrombosis. The incidence of complications in the OLIF group was 17.50%, which was lower than the 27.50% in the LLIF group, but the difference between the two groups was not statistically significant (χ²=1.147, P=0.284).

## DISCUSSION

The results of this study showed that at six months after surgery, the intervertebral space height and Cobb angle of the two groups of patients were significantly improved compared to before surgery (p<0.05), but there was no statistically significant difference between the groups (p>0.05). Both LLIF and OLIF surgical methods can restore intervertebral space height and correct the Cobb angle of the spine. This is mainly because before the implantation of the intervertebral fusion cage, both LLIF and OLIF surgeries can cut off the contralateral osteophyte, increase intervertebral mobility, and thus restore intervertebral space height and correct coronary deformities after the implantation of the intervertebral fusion cage.

Lumbar fusion technology is a commonly used method for treating degenerative diseases of the lumbar spine. Standard posterior or anterior approach opening or minimally invasive surgery can achieve a higher rate of interbody fusion.^11^ At present, LLIF/OLIF technology is an emerging minimally invasive surgery that can achieve good intervertebral fusion and correct deformities, with low surgical complications.^12^ The results of this study showed that the SF-36 score^10^, VAS score, ODI index, and JOA score of the two groups of patients were significantly improved after surgery (p<0.05), and pain was significantly relieved (p<0.05). In the short term, the improvement of SF-36 score, VAS score, ODI index, and JOA score in the OLIF group was better than that in the LLIF group (p<0.05), which may be due to frequent pain caused by continuous thigh numbness and lumbar muscle contracture in some patients in the LLIF group after surgery. However, there was a significant difference between the two groups of patients after long-term observation (p>0.05). The long-term efficacy of the two surgical methods, LLIF and OLIF, is basically equivalent. This is consistent with the results of multiple studies,^13^-^15^ indicating that the use of LLIF/OLIF in the treatment of lumbar degenerative diseases can achieve good clinical results. However, LLIF can cause damage to the lumbar muscles and large blood vessels.^16^ OLIF enters the target intervertebral disc space through the peritoneum and psoas major muscle space, without invading the psoas major muscle.^17^

However, there are literature reports that LLIF is superior to OLIF in correcting coronary segmental deformities and can effectively improve Cobb angle, while OLIF has a better ability to correct convex deformities than LLIF.^18^ This difference may be related to OLIF’s resection of the anterior longitudinal ligament. Related reports have shown that surgical complications have occurred in both LLIF and OLIF groups. In this study,^19^ both groups of patients also experienced complications, and the incidence was not significantly different (p>0.05), which is similar to the results reported in related studies.^20^

Osteoporosis or excessive intraoperative damage to the endplate can lead to serious complications such as fusion cage settlement or collapse. However, we did not observe fusion vessel settlement in our study, which may be related to our shorter follow-up time. Therefore, for patients with severe osteoporosis, the postoperative intervertebral fusion cage is prone to sinking, and it is not recommended to use LLIF/OLIF.^21^

### Limitations:

Due to the retrospective nature of this study, there are limitations such as a relatively small sample size of selected case data and a relatively short follow-up time. LLIF cannot be performed in patients who require posterior peritoneal surgery or have retroperitoneal abscesses or abnormal vascular anatomy.

## CONCLUSIONS

In conclusion, LLIF/OLIF surgical approaches should be considered safe and effective methods for treating isolated lumbar degenerative diseases. Both approaches may effectively improve patients’ clinical symptoms and radiographic manifestations, thereby enhance their quality of life.

### Authors’ Contributions:

**YW** and **HW:** Carried out the studies, participated in collecting data, and drafted the manuscript, and are responsible and accountable for the accuracy or integrity of the work. **YS** and **YM:** Performed the statistical analysis, Critical Review and participated in its design. **XS:** Performed the statistical analysis and participated in its design. And critical review. All authors read and approved the final manuscript.

## References

[1] Knezevic NN, Candido KD, Vlaeyen JWS, Van Zundert J, Cohen SP (2021). Low back pain. Lancet.

[2] Guo ML, Yue ST, Wang JY, Cui HX (2020). Comparative study on the clinical application of mixed reality technology leading micro-invasive intervertebral foramen puncture location and blind puncture location. Pak J Med Sci.

[3] Ouchida J, Kanemura T, Satake K, Nakashima H, Ishikawa Y, Imagama S (2020). Simultaneous single-position lateral interbody fusion and percutaneous pedicle screw fixation using O-arm-based navigation reduces the occupancy time of the operating room. Eur Spine J.

[4] Song M, Sun K, Li Z, Zong J, Tian X, Ma K (2021). Stress distribution of different lumbar posterior pedicle screw insertion techniques:a combination study of finite element analysis and biomechanical test. Sci Rep.

[5] Faur C, Patrascu JM, Haragus H, Anglitoiu B (2019). Correlation between multifidus fatty atrophy and lumbar disc degeneration in low back pain. BMC Musculoskelet Disord.

[6] Du X, Ou YS, Zhu Y, Luo W, Jiang GY, Jiang DM (2020). Oblique lateral interbody fusion combined percutaneous pedicle screw fixation in the surgical treatment of single-segment lumbar tuberculosis:A single-center retrospective comparative study. Int J Surg.

[7] Song QP, Hai B, Zhao WK, Huang X, Liu KX, Zhu B (2021). Full-Endoscopic Foraminotomy with a Novel Large Endoscopic Trephine for Severe Degenerative Lumbar Foraminal Stenosis at L5 S1 Level:An Advanced Surgical Technique. Orthop Surg.

[8] Fang X, Zhang M, Wang L, Hao Z (2022). Comparison of PLIF and TLIF in the Treatment of LDH Complicated with Spinal Stenosis. J Healthc Eng.

[9] Tu P, Cao S, Jiang C, Yan CC (2021). A comparative study of Lumbar Decompression and Fusion with Internal Fixation versus Simple Decompression in elderly patients with two-segment Lumbar Spinal Stenosis. Pak J Med Sci.

[10] Ware JE, Sherbourne CD (1992). The MOS 36-item short-form health survey (SF-36). I. Conceptual framework and item selection. Med Care.

[11] Mummaneni PV, Park P, Shaffrey CI, Wang MY, Uribe JS, Fessler RG (2019). The MISDEF2 algorithm:an updated algorithm for patient selection in minimally invasive deformity surgery. J Neurosurg Spine.

[12] Oda Y, Yamauchi T, Tanaka M (2019). Lateral Lumbar Interbody Fusion with Percutaneous Pedicle Screw in Combination with Microendoscopic Laminectomy in the Lateral Position for Lumbar Canal Stenosis. Acta Med Okayama.

[13] Wang W, Xiao B, Wang H, Qi J, Gu X, Ye X (2022). Oblique lateral interbody fusion stand-alone vs. combined with percutaneous pedicle screw fixation in the treatment of discogenic low back pain. Front Surg.

[14] Li J, Sun Y, Guo L, Zhang F, Ding W, Zhang W (2022). Efficacy and safety of a modified lateral lumbar interbody fusion in L4-5 lumbar degenerative diseases compared with traditional XLIF and OLIF:a retrospective cohort study of 156 cases. BMC Musculoskelet Disord.

[15] Mcgowan JE, Kanter AS (2019). Lateral approaches for the surgical treatment of lumbar spondylolisthesis. Neurosurg Clin N Am.

[16] Shen J, Zheng Q, Wang Y, Ying X (2021). One-stage combined anterior-posterior surgery for thoracic and lumbar spinal tuberculosis. J Spinal Cord Med.

[17] Kotani Y, Ikeura A, Saito T (2022). Comparative Clinical Analysis of Oblique Lateral Interbody Fusion at L5/S1 versus Minimally Invasive Transforaminal Interbody Fusion (MIS-TLIF) for Degenerative Lumbosacral Disorders. Spine Surg Relat Res.

[18] Chung HW, Lee HD, Jeon CH, Chung NS (2021). Comparison of surgical outcomes between oblique lateral interbody fusion (OLIF) and anterior lumbar interbody fusion (ALIF). Clin Neurol Neurosurg.

[19] Zhu L, Wang JW, Zhang L, Feng XM (2021). Outcomes of oblique lateral interbody fusion for adult spinal deformity:a systematic review and meta-analysis. Glob Spine J.

[20] Quillo-Olvera J, Lin GX, Jo HJ, Kim JS (2018). Complications on minimally invasive oblique lumbar interbody fusion at L2-L5 levels:a review of the literature and surgical strategies. Ann Transl Med.

[21] Ao S, Zheng W, Wu J, Tang Y, Zhang C, Zhou Y (2020). Comparison of Preliminary clinical outcomes between percutaneous endoscopic and minimally invasive transforaminal lumbar interbody fusion for lumbar degenerative diseases in a tertiary hospital:Is percutaneous endoscopic procedure superior to MIS-TLIF?A prospective cohort study. Int J Surg.

